# Editorial

**Published:** 1876-12

**Authors:** 


### Editorial.

Efficient sanitary regulations intelligently framed and fearlessly enforced should preserve the lives and health of the citizens of a great metropolis like our own. Under existing circumstances they are exposed to preventable diseases, and sacrificed to others, whose prevalence and severity could be greatly affected by an intelligent application of the established principles of sanitary science.

The sanitary system adopted in Chicago, is manifestly incomplete, and its administration utterly inefficient.

The Health Department of the city is little more than a bureau of registration of births and deaths; the registration itself being almost valueless from its want of accuracy. Beyond this, what does it practically accomplish? The accumulation of offal in the process of decomposition, by which the soil is saturated and the atmosphere vitiated, is permitted in the streets and alley-ways, and even in front of the homes of nearly half a million of people!

Already the results of this criminal negligence are to be observed. Diphtheria in a virulent form, and scarlatina of a malignant type are more or less prevalent in several districts.

If the present condition of affairs is allowed to continue unchanged, we may expect the appearance in our midst of other equally severe types of zymotic diseases.

Should the filth accumulations of to-day be suffered to remain, and become augmented by the usual increment of the winter, the ensuing warm season will but add to the noxious emanations from these hot beds of disease germs.

If the medical profession of the city shall not exert its influence, both in the creation of a proper public sentiment, and in the vehement urging of the municipal authorities to a more satisfactory performance of their public trust, certainly it will fall far short of its duty.

Only a false and mistaken economy will permit such grave consequences as are imminent, to occur. The judicious expenditure of a moderate sum of money at the present time, in an effort looking toward the removal of the sources of disease in Chicago, will not only prevent much needless suffering, but actually save many human lives.

